# Age-related reference intervals for ambulatory electrocardiographic parameters in healthy individuals

**DOI:** 10.3389/fcvm.2023.1099157

**Published:** 2023-03-06

**Authors:** Kenichi Hashimoto, Naomi Harada, Motohiro Kimata, Yusuke Kawamura, Naoya Fujita, Akinori Sekizawa, Yosuke Ono, Yasuhiro Obuchi, Tadateru Takayama, Yuji Kasamaki, Yuji Tanaka

**Affiliations:** ^1^ Department of General Medicine, National Defense Medical College, Tokorozawa, Saitama, Japan; ^2^ Department of Integrative Physiology and Bio-Nano Medicine, National Defense Medical College, Tokorozawa, Saitama, Japan; ^3^ Department of General Medicine, Nihon University School of Medicine, Tokyo, Japan; ^4^ Department of General Medicine, Kanazawa Medical College Himi Municipal Hospital, Himi, Toyama, Japan

**Keywords:** atrial fibrillation, amburatory ECG monitoring, premature ventricular complex (PVC), premature atrial complex (PAC), heart rate variability, Holter ECG monitoring

## Abstract

**Background:**

The advent of novel monitoring technologies has dramatically increased the use of ambulatory electrocardiography (AECG) devices. However, few studies have conducted detailed large-scale investigations on the incidence of arrhythmias over 24 h, especially ectopy, in healthy individuals over a wide age range.

**Objectives:**

This study aimed to investigate the incidence of arrhythmias detected using AECG and associated factors, in healthy individuals, over a wide age range.

**Methods:**

In this cross-sectional study, we performed AECG on 365 healthy volunteers (median [interquartile range]: 48 [36, 67], 20–89 years, 165 men) under free-running conditions for 24 h. Ultrasonic echocardiography and heart rate variability analysis were performed to explore the factors associated with the incidence of arrhythmias.

**Results:**

The 97.5th percentile of single ventricular ectopy (VE) was 149/day, 254/day, and 1,682/day in the 20–39-, 40–59- and 60–89-year age groups, respectively; that of single supraventricular ectopy (SVE) was 131/day, 232/day, and 1,063/day, respectively. Multivariate analysis revealed that aging was the only independent significant factor influencing the frequency of VE (*β* = 0.207, *P *= 0.001). Age (*β* = 0.642, *P *< 0.001), body mass index (BMI) (*β* = −0.112, *P *= 0.009), and the root mean square of successive differences in RR intervals (*β* = 0.097, *P *= 0.035) were factors significantly associated with SVE frequency.

**Conclusions:**

Age-specific reference intervals of VE and SVE in a large population of healthy participants over a wide age range were generated. VE and SVE increased with age; SVE was influenced by BMI and the aging-induced decrease in parasympathetic tone activity.

## 1. Introduction

The development of ambulatory electrocardiography (AECG) by Holter in 1957 enabled 24-h-ECG recording (1). Since then, AECG has been widely used for detecting arrhythmic events in clinical settings. Recently, the use of AECG devices has dramatically increased, especially with the advent of novel monitoring technologies, such as patch-type, implantable, and smartwatch-type ECG devices (2–4). Thus, it is necessary to establish reference intervals for AECG parameters to guide interpretation and clinical care. It is well-known that the prevalence of arrhythmic events depends on age. However, few studies have focused on the reference values for the prevalence of arrhythmias in each generation (younger, middle-aged, older populations) over a wide age range among healthy individuals. Previous studies on this subject included small sample sizes or were limited to fewer age groups, such as young (20–39 years) (5–9), middle-aged (40–59 years) (7, 10, 11), or older-aged cohorts (over 60 years) (7, 11–16). Moreover, most of these studies were conducted 20–40 years prior. Lifestyle and average longevity have changed over the 21st century, and few studies have investigated the incidence of arrhythmia in a wide age range using AECG.

Supraventricular ectopy (SVE) (incidence: 56%–87%) is reportedly the most common arrhythmia type in healthy individuals detected using AECG, followed by ventricular ectopy (VE) (incidence: 46%–69%) (17, 18). Previous studies have stated that SVE or VE should not be treated if they are infrequent or not severe in the absence of structural heart disease (19). However, recent studies have suggested that a higher frequency of ventricular extrasystole was associated with reversible cardiomyopathy (20), inducing a decreased left ventricular ejection fraction, increased chronic heart failure incidence, and a high mortality rate even in individuals without structural heart disease (21). Moreover, a recent study reported that frequent excessive supraventricular activity was associated with a risk of atrial fibrillation (AF), stroke, and total mortality in apparently healthy individuals (22). Therefore, establishing reference values of VE and SVE is of paramount importance. Furthermore, the factors influencing the incidence of VE and SVE are not fully understood.

This cross-sectional study entailed AECG examination of healthy volunteers whose ages varied widely, from 20 to 89 years. This study aimed to investigate the incidence of bradyarrhythmia and tachyarrhythmia and establish age-related reference values for AECG parameters. Moreover, we explored the factors associated with these AECG parameters, including ultrasonic echocardiography (UCG) and autonomic nervous system activity parameters expressed as heart rate variability (HRV), which can influence the prevalence of ectopy.

## 2. Materials and methods

### 2.1. Study population

We recruited healthy volunteers between April 2015 and March 2018 for this study. The inclusion criteria were as follows: individuals with no history of cardiovascular disease, respiratory disease, dyslipidemia, diabetes mellitus, chronic kidney disease, psychiatric disease, and autonomic nervous system disorders. Moreover, participants who underwent annual medical examinations within the past year without abnormal findings on chest radiographs and 12-lead ECG were also included. Night-shift workers and current smokers were excluded during the initial stage. A total of 400 participants, without structural heart disease, were initially included in this study. The study procedures included the following (in order): detailed medical history, general physical examination, systolic and diastolic blood pressure measurements, 12-lead standard ECG, ultrasonic echocardiology (UCG), and 24-h AECG. The recording time of AECG was stipulated to be more than 23 h/day. The exclusion criteria were as follows: participants with ST-T abnormalities on baseline 12-lead ECG, second- or third-degree atrio-ventricular (AV) block and left ventricular conduction block on standard 12-lead ECG, low ejection fraction (EF) (<50%) with wall motion abnormalities, significant left atrial dilatation and/or left ventricular dilatation detected with echocardiography, ST-T changes of an ischemic nature during daily activity and/or ambulatory ECG monitoring, long QT interval (>500 ms) on baseline 12-lead ECG, family history of sudden cardiac death, and body mass index (BMI) over 30 kg/m^2^.

Thirty-five participants [48 (36, 47)] were excluded from this study based on the above-mentioned exclusion criteria, while 365 participants were enrolled (Table 1). Most participants were healthy volunteers who were citizens of the Tokyo Metropolitan and Saitama Prefecture area, Japan. All volunteers provided written informed consent before participation. The study protocol conformed to the Declaration of Helsinki and was approved by the Medical Ethics Committee of the National Defense Medical College Hospital (approval no. 4645), Saitama, Japan, and Nihon University School of Medicine, Itabashi Hospital, Tokyo, Japan (approval no. MF 2208-0037).

**Table 1:** Demographics and Holter ECG and UCG results.

	20–39 years	40–59 years	60–89 years	*P*-value	Total
	(*n* = 120)	(*n* = 124)	(*n* = 121)		(*N* = 365)
Demographics
Age	31 [25, 36]	47 [43, 53]	71 [66.8, 75]	<0.001	48 [36, 67]
Men	61	52	53		166
Height (cm)	164.4 ± 9.2	162.8 ± 8.7	158.7 ± 9.2	<0.001	162.0 ± 9.3
Body weight (kg)	60.1 ± 10.8	62.2 ± 12.0	56.3 ± 9.9	<0.001	59.6 ± 11.2
Body mass index (kg/m^2^)	22.1 ± 4.1	23.3 ± 3.6	22.2 ± 2.5	0.016	22.6 ± 3.5
Systolic blood pressure (mmHg)	115.5 ± 11.4	126.2 ± 17.3	129.4 ± 14.2	<0.001	123.8 ± 15.7
Diastolic blood pressure (mmHg)	71.1 ± 10.4	79.4 ± 13.5	76.1 ± 10.9	<0.001	75.6 ± 12.2
AECG
Total beat/day	109,462.9 ± 12,341.5	110,534.9 ± 11,045.2	103,693.1 ± 11,055.1	<0.001	107,957.0 ± 1,1845.9
Maximum heart rate/day	144.0 ± 18.0	133.3 ± 14.7	121.1 ± 15.1	<0.001	131.0 [120.0, 143.0]
Minimum heart rate/day	50.6 ± 6.9	53.9 ± 6.2	53.0 ± 6.0	<0.001	52.5 ± 6.5
Mean heart rate/day	78.9 ± 8.7	78.8 ± 8.0	73.8 ± 8.1	<0.001	77.2 ± 8.6
Ventricular ectopy (single)/day	1.0 [0, 3.0]	2.0 [0, 6.0]	4.0 [0, 13]	<0.001	2.0 [0, 7]
Supra ventricular ectopy (single)/day	6.0 [2.0, 14]	13.0 [6.0, 30]	67.0 [30.0, 189]	<0.0001	18.0 [5.0, 51]
HRV
SDNN (ms)	154.7 [129.4, 186.3]	136.2 [115.8, 158.1]	129.9 [109.7, 153.2]	<0.001	139.5 [117.1, 165.6]
RMSSD (ms)	35.4 [27.1, 50.3]	25.0 [18.6, 32.5]	23.3 [17.1, 31.8]	<0.001	27.2 [20.6, 36.7]
pNN50 (%)	11.2 [5.2, 20.7]	4.0 [1.3, 9.0]	2.8 [0.8, 7.0]	<0.001	5.4 [1.9, 11.2]
SDANN (ms)	144.7 [113.8, 173.2]	125.4 [104.6, 146.2]	123.0 [99.8, 142.5]	<0.001	128.2 [107.4, 153.8]
VLF (ms^2^)	4,026.5[2,764.5, 6,373.8]	3,374.6 [2,424.0, 4,324.5]	2,996.0[2,155.8, 3,929.6]	<0.001	3,387.8[2,509.2, 4,741.7]
LF (ms^2^)	972.3 [698.9, 1,513.4]	586.2 [395.3, 885.4]	321.3 [214.5, 536.6]	<0.0001	616.6 [339.2, 1,001.9]
LFnu	17.1 [13.9, 20.0]	14.1 [10.8, 17.0]	9.4 [7.3, 12.5]	<0.0001	13.2 [9.9, 17.5]
HF (ms^2^)	418.3 [256.7, 900.5]	224.7 [118.3, 381.7]	152.8 [76.4, 259.6]	<0.0001	251.5 [129.1, 456.4]
HFnu	7.6 [5.4, 11.2]	4.9 [3.1, 7.6]	4.3 [2.6, 6.4]	<0.001	5.4 [3.4, 8.4]
LF/HF	2.2 [1.5, 2.8]	2.8 [1.9, 4.1]	2.2 [1.3, 3.2]	<0.001	2.3 [1.6, 3.4]
UCG
LVDd (mm)	46.8 ± 5.7	46.0 ± 6.2	45.9 ± 4.8	0.432	46.4 ± 5.7
EF (%)	67.4 ± 6.8	66.6 ± 6.3	67.1 ± 7.1	0.684	66.7 ± 6.9
E/e′ (septal)	5.8 ± 1.7	6.3 ± 1.8	6.6 ± 2.2	<0.001	6.1 ± 1.9
E/e′ (lateral)	4.9 ± 1.3	5.7 ± 1.6	5.9 ± 1.8	<0.001	5.4 ± 1.5
E/A	1.4 ± 0.4	1.2 ± 0.4	0.9 ± 0.3	<0.001	1.2 ± 0.4

EF, Left ventricular ejection fraction; E/e′, early diastolic flow velocity/ velocity of early diastolic mitral annular motion; HF, the power in the high-frequency area; HFnu, HF normalized unit; LF, low-frequency area; LF/HF, the power in the low-frequency/the power in the high-frequency ratio; LFnu, LF normalized unit; LVDd, left ventricular end-diastolic dimension; pNN50, percent of difference between adjacent normal; RR intervals greater than 50 ms, RMSSD (The square root of the mean of the sum of squares of differences between adjacent normal to normal intervals); SDANN, standard deviation of the 5-min average NN intervals; SDNN, standard deviation of the mean normal RR intervals for 5-min segments, ms; VLF, low frequency area; UCG, ultrasonic echocardiography.

### 2.2. Study protocol

Standard 12-lead ECG was performed, followed by UCG. Thereafter, an AECG recorder (FM180S, Fukuda Denshi Co., Ltd., Tokyo, Japan) was used to record the ECG for 24 h under free-running conditions, followed by analysis with the Holter ECG system (SCM8000, Fukuda Denshi Co., Ltd., Tokyo, Japan).

### 2.3. Routine AECG data analysis

Routine AECG data analysis was performed automatically with manual editing (Table 1). The parameters analyzed included the total number of beats, maximum, minimum, and mean heart rate (HR), and the frequency of VE and SVE per day. Ventricular arrhythmias were defined as follows: ventricular tachycardia (VT), ≥3 consecutive ventricular complexes at a rate >100 bpm; ventricular triplet (V3), more than three ventricular ectopic beats in a row at a rate <100 bpm; and ventricular couplet (V2), two ventricular ectopic beats in a row. Supraventricular arrhythmia was defined as follows: supraventricular tachycardia (SVT), ≥3 consecutive ventricular complexes at a rate >150 bpm; supraventricular triplet (S3), more than three supraventricular ectopic beats in a row at a rate <150 bpm; and supraventricular couplet (S2), two supraventricular ectopic beats in a row. The total number of beats, and the maximum and mean HR were significantly lower in the older generation (*P* < 0.001 for all, respectively) (Table 1). Thus, the prevalence of VE and SVE was significantly higher in the older-aged group (*P* < 0.001 and *P* < 0.0001, respectively) (Table 1).

### 2.4. Analysis of HRV

HRV analysis was also performed to evaluate autonomic nervous system activity using the SCM 8,000 system (Fukuda Denshi Co., Ltd., Tokyo, Japan) (Table 1). The RR interval was calculated for HRV analysis *via* the corrected maximum entropy method using Akaike's algorithm, as previously reported (23). The HRV data were subjected to time and frequency domain analyses at 60-min intervals. The definitions of all HRV parameters were based on previous studies (24). The parameters for time domain analysis, which were evaluated every 5 min over 24 h, included the following: standard deviation of the mean normal RR interval (SDNN), the square root of the mean of the sum of the squares of differences between adjacent normal to normal intervals (RMSSD), proportion of times between adjacent cycles that are different by >50 ms (pNN50), and standard deviation of the averages of NN intervals in all 5-min segments of the entire recording (SDANN). Frequency domain analysis entailed evaluation of the power in the very low-frequency area (VLF), power in low-frequency area (LF), power in the high-frequency area (HF), and power in the low-frequency/power in the high-frequency (LF/HF) ratio every 5 min. The power spectra of frequency domain analysis were defined as follows: total power (TP), approximately <0.4 Hz; power in the very low-frequency range (VLF), 0–0.04 Hz; power in the low-frequency range (LF), 0.04–0.15 Hz; and power in the high-frequency range (HF), 0.15–0.40 Hz. The normalized values (nu) were calculated using the following formula: LF/TP × 100 for LFnu, and HF/TP × 100 for HFnu. All HRV parameters, except for LF/HF, were significantly lower in the older generation (*P *< 0.001 for SDNN, RMSSD, pNN 50, SDANN, VLF; *P *< 0.0001 for LF, HF, and HFnu) (Table 1).

### 2.5. Echocardiography recordings

Echocardiography was performed using the Xalio (Toshiba Co., Ltd., Tokyo, Japan) system to evaluate left ventricular EF and left ventricular end-diastolic dimension (LVDd). Left ventricular EF was calculated during sinus beats using Simpson's method (25). LVDd and EF did not differ significantly among the three generations (*P *= 0.432 and *P *= 0.684). E/e′ (septal) and E/e′ (lateral) were significantly higher in the older generation (*P *< 0.001 for all, respectively) (Table 1).

### 2.6. Statistical analyses

Data are presented as the mean ± standard deviation for normally distributed continuous variables, and as medians (interquartile range: 25–75th percentile) for non-normally distributed variables. Patient characteristics including demographic features, and the AECG, HRV, and UCG parameters were compared using the *χ*^2^ test for categorical variables, analysis of variance for continuous and parametric data, and the Kruskal–Wallis test for nonparametric data. Comparisons of frequencies among each hour in bradyarrythmias (sinus pause and AV block) and VE/SVE were performed using the Kruskal–Wallis test. The parameters influencing the ectopy prevalence in each generation were also compared using the Kruskal–Wallis test; *post hoc* multiple comparisons were performed using the Bonferroni method.

Multivariate regression analysis was performed to determine the intensity of the incidence of premature atrial and ventricular complex and theoretical consideration of important factors such as the UCG and HRV indices. We also selected age, sex, and BMI as the explanatory variables for multivariate analysis. Before performing multiple regression for the incidence of VE and SVE, the HRV indices (SDNN, SDANN, RMSSD, PNN50, LFnu, HFnu, and LF/HF) were transformed to natural logarithms, as these parameters showed skewed distributions. Multivariate linear regression was performed after simultaneously controlling for potential confounders, followed by step-wise selection or backward selection. Log SDANN and log LFnu were excluded owing to multicollinearity (variance inflation factor >10) during multivariate regression analysis for both VE and SVE. We set the reference interval for the AECG parameters as the 2.5th–97.5th percentile according to the Clinical and Laboratory Standards Institute guidelines and meta-analysis (26, 27). Furthermore, the sample size of the reference interval of AECG parameters was set at 120 participants minimum in each generation (20–39, 40–59, and 60–89 years) according to the Clinical and Laboratory Standards Institute guidelines (26). Statistical analyses were conducted using SPSS version 28 (IBM Corp, Armonk, NY, USA). All tests were two-sided, and *P*-values <0.05 were considered statistically significant.

## 3. Results

### 3.1. Sinus pauses and conduction abnormalities

**Figure 1:**
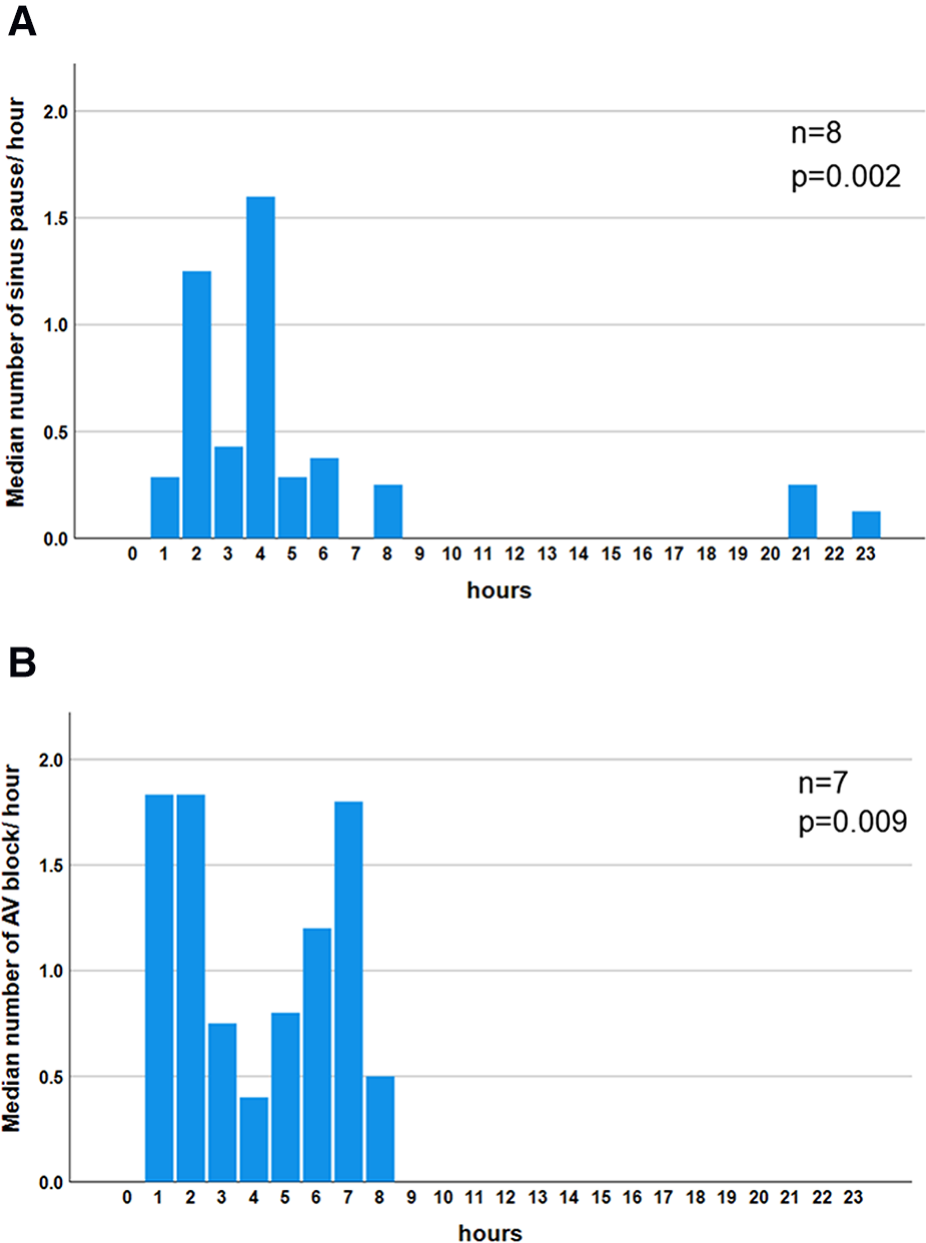
Diurnal variation in the median number of bradyarrhythmias. Significant night-time dominance was observed in the diurnal variation of the median number of sinus pauses (*P *= 0.002) (**A**). Significant night-time dominance in the diurnal variation of the median number of atrio-ventricular blocks (*P *= 0.009) (**B**). Comparisons of frequencies among each hour in sinus pause and AV block were performed using the Kruskal–Wallis test. AV block: atrio-ventricular block.

The incidence of sinus pause >2 s was 4.1%, 1.6%, and 0.8% in the 20–39-, 40–59-, and 60–89-year age groups, respectively (Table 2). The incidence was higher in the younger-aged group. The incidence of pause >2 s was under 5% in all generations, rendering these findings abnormal. Generation-dependent incidence was observed in the case of second-degree AV block, akin to sinus pause. Additionally, the incidence of second-degree AV block (i.e., abnormal findings) was under 5% for all generations, rendering these findings abnormal. The evaluation of the diurnal variations in the prevalence of sinus pause and second-degree AV block revealed that both were observed mainly at night-time: from 21:00 to 8:00 (Figures 1A,B).

**Table 2:** Complex premature beats on 24-h AECG (*N* = 365).

	Sinus pauses >2 s	Sinus pauses >3 s	Second-degree AV block (Wenckebach)	Second-degree AV block (Mobitz)	Third-degree AV block
20–39 years (*n* = 120)	5 (4.1%)	0 (0%)	4 (3.3%)	0 (0%)	0 (0%)
40–59 years (*n* = 124)	2 (1.6%)	0 (0%)	2 (1.6%)	0 (0%)	0 (0%)
60–89 years (*n* = 121)	1 (0.8%)	0 (0%)	1 (0.8%)	1 (0.8%)	0 (0%)
20–89 years (*n* = 365)	8 (2.1%)	0 (0%)	7 (1.9%)	1 (0.2%)	0 (0%)

AECG, ambulatory electrocardiography; AV, atrioventricular.

### 3.2. Percentile of simple VE and SVE number (reference values of ectopy)

The principal results of this study are presented in Table 3 and summarized in the Figures 3A,B. The 97.5th percentile of simple VE frequency (reference values of ectopy) was 149, 254, and 1,682/day in the 20–39-, 40–59-, and 60–89-years age groups, respectively. The overall reference value for premature ventricular ectopy for all generations was 366/day. On the other hand, the 95th percentile of the frequency of simple SVE (reference values of extrasystole) was 131, 232, and 1,063/day for the 20–39-, 40–59-, and 60–89-year age groups, respectively. Overall, the reference value of SVE for all generations was 537/day.

**Table 3:** Percentile of the frequency of simple ectopy (reference value of ectopy) (*N* = 365).

	Percentile								
	2.5	5	10	25	50	75	90	95	97.5
VE
20–39 years	0	0	0	0	1	3	9	59	149
40–59 years	0	0	0	0	2	6	37	144	254
60–89 years	0	0	0	1	4	13	171	393	1,682
20–89 years	0	0	0	0	2	7	57	194	366
	Percentile								
	2.5	5	10	25	50	75	90	95	97.5
SVE									
20–39 years	0	0	0	2	6	13	24	52	131
40–59 years	1	1	2	6	13	29	55	125	232
60–89 years	4	9	14	27	67	189	453	558	1,063
20–89 years	0	0	2	5	18	50	186	311	537

SVE, supraventricular ectopy; VE, ventricular ectopy.

**Figure 2:**
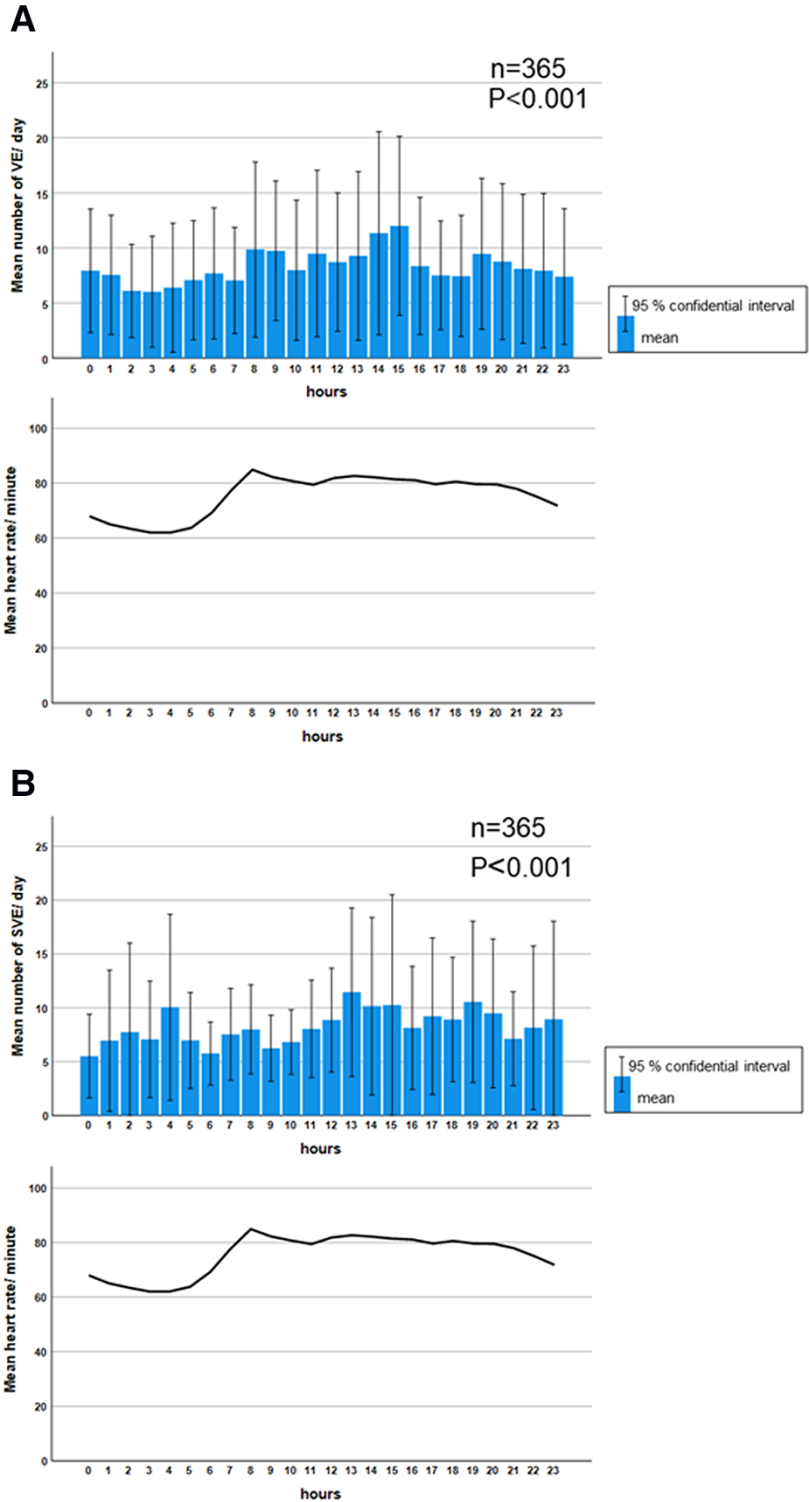
Diurnal variation in the median frequency of ventricular and supraventricular ectopy. A significant diurnal variation was observed in the mean frequency of VE (*P *< 0.001). The daytime prevalence of VE was predominant, which was parallel to the diurnal variation in HR (**A**). Meanwhile, the mean frequency of SVE was significantly higher at 4:00 and during 13:00–15:00 (*P *< 0.001). However, the diurnal variation of SVE was not parallel to the diurnal variation of HR (**B**). Comparisons of frequencies among each hour in VE and SVE were performed using the Kruskal–Wallis test. HR: heart rate, SVE: supraventricular ectopy, VE: ventricular ectopy.

Significant diurnal variation was observed in the mean HR and mean frequency of VE and SVE. The mean frequency of VE was significantly higher during the waking hours (8:00–24:00) than during sleeping hours (23:00–7:00) (*P *< 0.001) (Figure 2A). In contrast, the mean frequency distribution of SVE had two peaks at 4:00 and 13:00–15:00 (*P *< 0.001) (Figure 2B).

### 3.3. Complex VE and atrial SVE

The findings associated with complex ectopy and tachycardia are described in Table 4. VE Multiform was observed in 138/365 (37.8%) of the participants (Table 4). VT and V3 were observed in 6/365 (1.6%) and 4/365 (1%) of the participants, respectively, whereas R-on-T were not observed in any participant. All types of SVT, S3, and S2 were observed in 37/365 (10.1%), 86/365 (23.5%), and 151/365 (41.3%) participants, respectively. The incidence of complex SVE increased with age progression (Table 4).

**Table 4:** Complex ectopy and tachycardia on 24-h AECG (*N* = 365).

VE	Incidence	Multiform	VT	V3	V2	R-on-T	Bigeminy	Trigeminy
20–39 years (*n* = 120)	68 (56.7%)	27 (22.5%)	2 (1.7%)	0(0%)	2 (1.6%)	0 (0%)	4 (3.3%)	2 (1.7%)
40–59 years (*n* = 124)	88 (71.0%)	46 (37.0%)	2 (1.6%)	0 (0%)	6 (4.8%)	0 (0%)	4 (3.2%)	2 (1.6%)
60–89 years (*n* = 121)	102 (84.3%)	65 (53.7%)	2 (1.7%)	4 (3.3%)	16 (13.2%)	0 (0%)	10 (8.2%)	11 (9%)
20–89 years (*N* = 365)	258 (70.7%)	138 (37.8%)	6 (1.6%)	4 (1%)	25 (6.6%)	0 (0%)	18 (4.9%)	15 (4.1%)
SVE	Incidence	All types of SVT	SVT >10 beats	S3	S2			
20–39 years (*n* = 120)	103 (85.8%)	1 (0.8%)	0 (0%)	6 (5%)	19 (15.8%)			
40–59 years (*n* = 124)	122 (98.4%)	4 (3.2%)	0 (0%)	22 (17.7%)	42 (33.9%)			
60–89 years (*n* = 121)	121 (100%)	32 (26.4%)	10 (12%)	73 (60.3%)	90 (74.3%)			
20–89 years (*N* = 365)	346 (94.7%)	37 (10.1%)	10 (2.7%)	86 (23.5%)	151 (41.3%)			

### 3.4. Correlation between the incidence of ectopy and UCG and HRV indices

**Figure 3:**
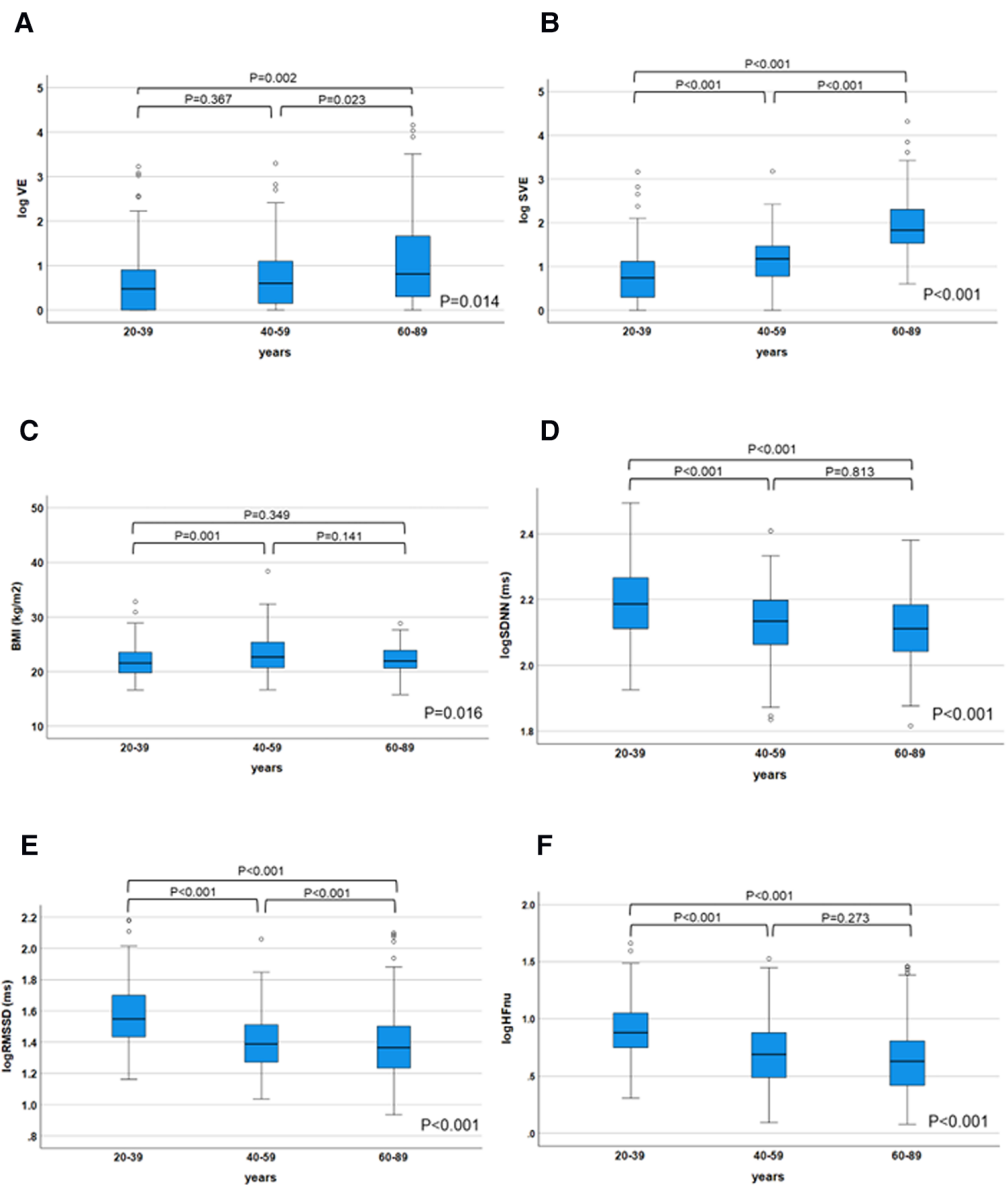
Changes in the ectopy prevalence and parameters influencing ectopy prevalence in each generation. The log VE/day and log SVE/day were significantly decreased in the older generation (*P *= 0.014 for log VE; *P *< 0.001 for log SVE), (**A**), (**B**). On the other hand, body mass index was significantly higher in the 40–59-year age group than in the 20–39- and 60–89-year age groups (*P *= 0.016) (**C**). HRV parameters (log SDNN, log RMSSD, and log HFnu), which influenced the prevalence of SVE, were significantly decreased in the older generation (*P *< 0.001 for all, respectively) (**D–F**). The parameters influencing the ectopy prevalence in each generation were compared using the Kruskal–Wallis test; *post hoc* multiple comparisons were performed using the Bonferroni method. HRV, heart rate variability; SVE, supraventricular ectopy; VE, ventricular ectopy.

Multivariate regression analysis was performed to explore the intensity of factors affecting the incidence of VE and SVE. Log VE was higher in the older generation (*P *= 0.014) (Figure 3A). Age was an independent factor influencing the VE incidence (*β* = 0.293, *P *= 0.001), whose effect was retained in step-wise selection (*β* = 0.207, *P *= 0.001) (Table 5). In a sub-analysis, multivariate regression analysis with the backward selection method showed that age tended to be the most influential factor for log VE in all the generations (20–39, 40–59, and 60–89 years) (*P* = 0.054–0.079) (Supplementary Tables S1–S3). Log SVE was higher in the older generation (*P *< 0.001) (Figure 3B). However, BMI was significantly higher in the 40–59-year age group than in the 20–39- and 60–89-year age groups (*P* = 0.016) (Figure 3C). Hence, log SDNN, log RMSSD, and log HFnu were lower in the older generation (*P *< 0.001 for all) (Figure 3D,E,, F). Age, BMI, log SDNN, log RMSSD, and log HFnu were significant factors affecting the SVE incidence (age, *β* = 0.532, *P *< 0.001; BMI, *β* = −0.099, *P *= 0.029; log SDNN, *β* = −0.136, *P *= 0.02; RMSSD, *β* = 0.457, *P *< 0.001; log HFnu, *β* = −0.368, *P *= 0.001). Moreover, these indices were significant factors affecting the SVE incidence, even according to step-wise selection (age, *β* = 0.642, *P *< 0.001; BMI, *β* = v0.112, *P *= 0.009; log RMSSD, *β* = 0.097, *P *= 0.035) (Table 6). In contrast, a sub-analysis was performed on the most influential factors for log SVE in each generation (20–39, 40–59, and 60–89 years). Multivariate analysis revealed that age, BMI, and RMSSD were significant factors (Supplementary Tables S4–S6), with a similar trend as in the analysis of all generations (Table 6). However, in the 60–89-year age group, BMI was not a significant factor.

**Table 5:** Multiple regression analysis for log ventricular ectopy (*n* = 365).

	*R* = 0.324			*R* = 0.207^a^		
	*β*	*P*	VIF	*β*	*P*	VIF
log VE	-	-	-	-	-	-
Age	0.293	0.001	2.08	0.207	0.001	1.0
Sex	−0.044	0.532	1.274			
SBP	−0.133	0.064	1.338			
BMI	0.067	0.315	1.161			
E/e′ (septal)	0.139	0.183	2.856			
E/e′ (lateral)	0.019	0.852	2.88			
E/A	0.089	0.217	1.353			
EF	0.033	0.596	1.041			
logSDNN	−0.153	0.074	1.92			
logRMSSD	0.041	0.827	9.336			
logPNN50	0.085	0.557	5.467			
logHFnu	0.081	0.62	7.045			
log LF/HF	0.108	0.367	3.795			

EF, left ventricular ejection fraction; HF, power in the high-frequency area; HFnu, HF normalized unit; LF, low-frequency area; LF/HF, power in the low-frequency/power in the high-frequency ratio; LFnu, LF normalized unit; LVDd, left ventricular end-diastolic dimension; pNN50,percent of difference between adjacent normal RR intervals greater than 50 ms; RMSSD, root mean square successive difference; SBP, systolic blood pressure; SDNN, standard deviation of the mean normal RR intervals for 5-min segments (ms); VIF, variance inflation factor; VLF, low frequency area. a Varibles by multiple linear regression with stepwise selection.

**Table 6:** Multiple regression analysis for log supraventricular ectopy (*N* = 365).

	R = 0.653			R = 0.626^a^		
	*β*	*P*	VIF	*β*	*P*	VIF
log SVE	-	-	-	-	-	-
Age	0.532	<0.001	2.235	0.642	<0.001	1.15
Sex	−0.003	0.946	1.308			
SBP	−0.028	0.561	1.397			
BMI	−0.099	0.029	1.191	−0.112	0.009	1.03
E/e′ (septal)	0.071	0.309	2.832			
E/e′ (lateral)	0.012	0.866	2.883			
E/A	0.005	0.923	1.362			
EF	0.031	0.459	1.031			
logSDNN	−0.136	0.02	2.001			
logRMSSD	0.457	<0.001	8.828	0.097	0.035	1.18
logPNN50	−0.136	0.168	5.665			
logHFnu	−0.368	0.001	7.497			
logLF/HF	−0.164	0.051	4.09			

log SVE, log supra ventricular ectopy; the other abbreviations as in Table 5.

^a^ Variables by multiple linear regression with stepwise selection.

## 4. Discussion

In this study, we provided age-specific reference values for AECG parameters, including bradycardia detected using 24-h AECG, in each generation, spread over a wide age range (20–89 years) in a healthy population. Moreover, we provided evidence that the incidence of VE was only related to the increase in age; hence, SVE was influenced by the increase in age and BMI and decrement in RMSSD and HFnu, which are reflective of parasympathetic nervous system activity. This is the first study to demonstrate the relationship between autonomic tone activity, expressed as HRV, and the incidence of VE and SVE over a wide age range.

### 4.1. Reference intervals of AECG parameters

The differences between the “normal” and “abnormal” AECG findings in each generation (20–39, 40–59, and 60–89 years) (Table 7) were determined, based on the assumption that events occurring in less than 2.5% of a healthy population were “abnormal” and those occurring in more than 2.5% of the population were “normal.” The 2.5th–97.5th range is defined as the reference interval in the Clinical and Laboratory Standards Institute guidelines, as well as in many of the articles included in the meta-analysis and the meta-analysis itself (26, 27). Therefore, in the present study, the 95th percentile distribution was also defined as the reference interval or reference value. The strength of the reference values calculated in our study is the mild skew in age and sex, and the wide age range (20–89 years) of the participants (Table 1).

**Table 7:** Differentiation between the normal and abnormal AECG findings in each generation.

	Normal	Abnormal
20–39 years (*n* = 120)
Bradycardia	Sinus pause <3 s	Sinus pause >3 s, Second-degree atrio-ventricular block (Mobitz), Third-degree atrio-ventricular block
Ectopy and tachycardia (ventricular)	VE < 149	VE >149, Multiform VE, VT, V3, V2, R on T, Bigeminy, Trigeminy
Ectopy and tachycardia (supraventricular)	SVE <131, S3, S2	SVE >131, any SVT
40–59 years (*n* = 124)
Bradycardia	Sinus pause <2 s	Sinus pause >3 s, All second-degree atrio-ventricular block, Third-degree atrio-ventricular block
Ectopy and tachycardia (ventricular)	VE <232	VE >232, Multiform VE, VT, V3, V2, R on T, Bigeminy, Trigeminy
Ectopy and tachycardia (supraventricular)	SVE <144, S3, S2	SVE >144 SVT >10 beat
60–89 years (*n* = 121)
Bradycardia	Sinus pause <2 s	Sinus pause >3 s, All second-degree atrio-ventricular block, Third-degree atrio-ventricular block
Ectopies and tachycardia (ventricular)	VE <1,682, V2, V3, Bigeminy, Trigeminy	VE >1,682, Multiform VE, VT, R on T
Ectopy and tachycardia (supraventricular)	SVE <1,063, S3, S2	SVE >1,063 SVT >10 beat

AECG, ambulatory electrocardiogram; S2, supraventricular couplet S3, supraventricular triplet; SVE, supra ventricular ectopy; SVT, supraventricular ventricular tachycardia; V2, ventricular couplet; V3, ventricular triplet; VE, ventricular ectopy; VT, ventricular tachycardia.

Moreover, we set stringent criteria for healthy participants in this study, who were defined as individuals with no history of the following: cardiac abnormalities, abnormality on physical examination, 12-lead ECG, chest radiograph, blood investigations, and almost normal UCG findings; previous studies did not establish such strict criteria, especially with respect to blood work and UCG (25). Therefore, it is possible to designate this result as a precise reference interval. However, this reference interval is not normally distributed. There is a large discrepancy in the 90–97.5 percentile, especially in VE and SVE; therefore, this value should be treated with caution (Table 3). Since strict criteria of reference values, such as those in this study, have not existed in recent years, this information may be very useful not only for physicians but also for healthcare professionals in clinical settings in many situations, such as outpatient clinics and health checkup posts. Moreover, this reference interval could be versatile, because an AECG is performed in not only cardiology, but also various other medical departments and in general medicine.

### 4.2. Sinus pauses and conduction abnormality

We found that the incidence of sinus pause >3 s and second-degree AV block (Mobitz)was less than 2.5% in all generations (Table 2). Therefore, sinus pause >3 s and second-degree AV block (Mobitz) are abnormal findings in healthy individuals. Although the incidence of

second-degree AV block (Wenckebach) in 20–39 years was more than 2.5%, that in 40–59 and 60–89 years was less than 2.5% each. Therefore, second-degree AV block (Wenckebach) in an abnormal finding in 40–59 and 60–89 years. Moreover, the incidence of bradyarrhythmia was higher in the younger-age group. These findings are consistent with those of a previous meta-analysis (27). Hingorani et al. reported that the incidence of sinus pause >2 s in 1,273 healthy normal volunteers aged 18–45 and 46–65 years was 4.4% and 0%, respectively, whereas the incidence of second-degree AV block was 2.6% and 0.9%, respectively (17). The precise pathogenesis responsible for the higher incidence of bradyarrhythmia in the younger population is unknown. However, we speculated that autonomic nervous system activity, especially parasympathetic dominance, in younger individuals contributes to the susceptibility to bradyarrhythmia. The night-time predominance of sinus pause and diurnal variation in the AV block suggests the involvement of parasympathetic tone in these arrhythmias. Our findings provide valuable evidence, as no study has focused on diurnal variation in bradycardia detected on AECG (27).

### 4.3. Reference intervals of VE and SVE

We provided reference values for both VE and SVE in each generation (20–39, 40–59, and 60–89 years) over a wide age range in a population (Table 3 and central illustration). Several studies have reported on the frequency of VE and SVE/24 h using AECG in a few age groups in apparently healthy participants (Tables 8–10) (5–16). However, few studies have demonstrated reference values in each generation (20–39, 40–59, and 60–89 years) over a wide age range. Notably, the frequency of VE and SVE/24 h was higher in the older-aged group than in the younger-aged group in all percentile categories (from the 2.5th–97.5th percentiles) (Table 3). Recently, Williams et al. conducted a meta-analysis of 33 studies from 1950 to 2020 concerning reference intervals for AECG parameters and reported that the normal range of VE and SVE was 0–500/24 h, 0–1,000/24 h, and >1,000/24 h, for 20–39, 40–59, and 60–89 years, respectively (27). These findings are consistent with our data, except for the older generation (60–89 years). However, most studies (28 of 33) incorporated in that meta-analysis were published before 2000. The reference values for the young generation (18–36 years) were published in 1981 (Table 8). Moreover, reference values for the middle-age group are lacking (Table 9). Notably, the latest study to report reference values (age range: 64–80 years) of VE and SVE in a Japanese population was published in 2006, but data for establishing the reference values were collected in 1989 (Table 10) (15). Thus, the data used, and inferences derived from these studies are extremely old. Therefore, our study's findings significantly contribute to and expand the existing body of evidence. We also investigated complex ectopy and tachycardia using 24-h AECG (Table 4). The incidence of VT, R-on-T, and SVT >10 beats in 20–39 and 40–59 years generation were less than 2.5% in all generations; i.e., these findings are abnormal in healthy individuals. However, the prevalence of bigeminy and trigeminy in the 60–89 years age group was 8.2% and 9%, respectively. To the best of our knowledge, this study was the first to conduct such a detailed analysis, rendering these findings novel.

**Table 8:** Prior study of reference values of VE and SVE in healthy subjects (20–39 years).

				VE			SVE						
Author	Age	Subject No	Men (%)	90 percentile	95 percentile	97.5 percentile	90 percentile	95 percentile	97.5 percentile	Screening	Year	Race	Ref no
Brodsky	23–27	50	50 (100%)	10 (88 percentile)	-	>50 (98 percentile)	10 (92 percentile)	-	>100 (98 percentile)	PH, PE, 12-ECG, CXR, UCG	1977	USA	5
Okajima	18–36	102	56 (55%)	-	100 (96 percentile)	-	-	100 (96 percentile)	-	PH, PE, 12-ECG	1981	Japanese	6
Ramusen	20–39	36	19 (53%)	-	35	-	-	-	-	PH, PE, 12-ECG, BW	1985	Denmark	7
Gomer	15–39	43	43 (100)	10	70	-	14	504	-	PH,12-ECG, PE	1986	Sweden	8
Von Rotz	25–41	2,043	953 (47%)	-	193	-	-	-	-	PH, PE, BW	2017	Liechten-stein	9
Hashimoto (This study)	20–39	120	61 (52%)	9	59	149	24	52	131	PH, PE, 12-ECG, CXR, UCG, BW	2022	Japanese	

BW, blood work; CXR, Chest x-ray; PE, Physical examination; PH, past history; 12-ECG, 12 leads electrocardiogram; UCG, ultrasonic echocardiography.

**Table 9:** Prior study of reference values of VE and SVE in healthy subjects (40–59 years).

				VE			SVE						
Author	Age	Subject No	Men (%)	90 percentile	95 percentile	97.5 percentile	90 percentile	95 percentile	97.5 percentile	Screening	Year	Race	Ref no
Kostis	48 ± 10	101	51 (50%)	100	-	-	-	-	-	PH, PE, 12-ECG, CXR, UCG, BW, EST, CAG	1981	USA	10
Bjerregaard	40–59	184	126 (68%)	100–200	-	200–1,000 (97.8 percentile)	-	-	10–100 (97.8 percentile)	PH, PE, 12-ECG, CXR	1982	Denmark	11
Gomer	40–66	43	43 (100)	100–200	300	-	-	-	-	PH, 12-ECG, PE	1986	Sweden	8
Ramusen	30–49	38	19 (50%)	233	864	-	156	461	-	PH, PE, 12-ECG, BW	1985	Denmark	7
Hashimoto (This study)	20–49	124	52 (42%)	37	144	254	55	125	232	PH, PE, 12-ECG, CXR, UCG, BW	2022	Japanese	

BW, blood work; CXR, Chest x-ray; EST, (maximal) exercise stress test; CAG, coronary angiography; PE, Physical examination; PH, past history; 12-ECG, 12 leads electrocardiogram; UCG, ultrasonic echocardiography.

**Table 10:** Prior study of reference values of VE and SVE in healthy subjects (>60 years).

				VE			SVE						
Author	Age	Subject No	Men (%)	90 percentile	95 percentile	97.5 percentile	90 percentile	95 percentile	97.5 percentile	Screening	Year	Race	Ref no
Bjerregaard	60–79	76	44 (59%)	200–1,000	-	>1,000	10–100	>100	-	PH, PE, 12-ECG, CXR	1982	Denmark	11
Ramusen	60–79	37	19 (51%)	300–400	500	-	-	-	-	PH, PE, 12-ECG, BW	1985	Denmark	7
Kantelip	80–100	44	6 (14%)	1,200–2,400	2,400 (96 percentile)	-	1,200–2,400	-	2,400 (98 percentile)	PH, PE, 12-ECG, BW	1986	France	12
Anderson	73, 82	32	16 (50%)	<1,000	-	-	<1,000	-	-	PH, PE, 12-ECG	1988	Sweden	13
Ribera	58–85	50	30 (60%)	-	139	-	-	298	-	PH, PE	1989	Spain	14
Tasaki	64–80	15	5 (33%)	-	69	-	-	419	-	PH, PE, 12-ECG, CXR, BW	1989^a^	Japanese	15
Hashimoto (This study)	60–89	121	53 (44%)	171	393	1,682	453	558	1,063	PH, PE, 12-ECG, CXR, UCG, BW	2022	Japanese	

BW, blood work’ C-XR, Chest x–ray; PE, Physical examination; PH, past history; 12-ECG, 12 leads electrocardiogram; UCG, ultrasonic echocardiography.

^a^ The paper reported by Tasaki et, was published in 2000, but the data of AECG was detected in 1989.

### 4.4. Correlation between ventricular ectopy and UCG or HRV parameters

In the present study, VEs were only correlated with age, whereas SVEs were correlated with BMI, age, log RMSSD, and log HFnu. Aging has the greatest influence on the frequency of VE and SVE. Regarding VE, age was the independent factor that affected the number of VE through all the generations. VE had no relationship with the other factors in Figures 3D–F (log SDNN, log RMSSD, and log HFnu). In the sub-analysis, age tended to be the most influential factor affecting VE, although it was not statistically significant (Supplementary -Tables S1–S3). We speculate that this may have been the case because the sub-analysis was divided based on the generations and therefore did not reach significance. It has been widely reported that the prevalence of VE in the older population was higher than that in the younger population, which was also proven in a meta-analysis (27). Moreover, Tasaki et al. followed a cohort of healthy individuals for 15 years and found that the incidence of VE and SVE increased significantly after 15 years (15). Therefore, although the higher incidence of VE and SVE in older individuals is an unquestionable fact, few studies have investigated the mechanism of this phenomenon. The age-related changes in intracellular Ca^2+^ regulation which play an important role in the development of several types of arrhythmias may explain this phenomenon (28). Studies have suggested that age-related changes in intracellular Ca^2+^ regulation may prolong the action potential, especially during tachycardia, inducing electrical instability due to inadequate return of intracellular Ca^2+^ concentration. VE, which accounts for the high diurnal variation in VE when HR is elevated during the day, supports this hypothesis.

### 4.5. Correlation between supraventricular ectopy and UCG or HRV parameters

Several studies have reported that the frequency of SVEs increases with age, but few have examined the correlation between the frequency of ectopy (SVE) and UCG and HRV parameters simultaneously. SVE was inversely correlated with parasympathetic indices such as log RMSDD and log Hfnu (Figures 3B,E,F), thereby supporting the results of the multivariate analysis in Table 6. In contrast, age, obesity, and RMSSD, a parasympathetic index, were significant factors influencing SVE, as was the case for all age groups (Table 6 and Supplementary Tables S4, S5). However, BMI was not significant in the 60–89-year age group (Supplementary Table S6). The possible causes for this are as follows: BMI was lower in this generation than in the 40–59-year age group (Figure 3C) and the small variation in BMI made it less likely to be statistically significant.

The causes for the increase in frequency of SVE with age have not been clarified in humans; however, the following speculations are made regarding the basic experimental study. Age-related changes in ion channels in the atria and ventricles are key to the dynamics of Ca^2+^ channels. In the animal experimental study, the uptake of Ca^2+^ into the sarcoplasmic reticulum decreases with age and intracellular Ca^2+^ increases with age (29). Increased intracellular Ca^2+^ causes early posterior depolarization and induces APC and AF (30). Conversely, it has been reported that aging (degree of frailty) correlates with prolongation of the *P* wave and PR interval in ECGs of aged mice and that this is caused by elevated levels of interstitial fibrosis and collagen content (31). The above structural remodeling has been reported to increase the frequency of AF from APCs with aging.

It is generally recognized that RMSSD, pNN50, and HFnu are parameters related to parasympathetic nervous system activity (24). Therefore, there is a possibility that the increment in the frequency of SVE with aging partially results from decreased autonomic nervous system activity due to aging, particularly parasympathetic nervous system activity. Automaticity or triggered activity is thought to be the mechanism underlying SVE occurrence (32). It is speculated that a decrease in parasympathetic activity can lead to an increase in automaticity (32), which may be responsible for the decrease in parasympathetic activity in middle-aged and older individuals and may increase the frequency of SVEs with aging. It has been widely reported that the incidence of AF increases in middle-aged and older individuals (33). The incidence of SVE due to aging and the change in the equilibrium of sympathetic/parasympathetic activity may influence the increase in AF in older individuals. Incidentally, fluctuations in heart rate variability, expressed as SDNN, became significantly smaller with age. This result is consistent with that of previous reports and is an age-related change (34).

In this study, multiple regression analysis revealed that BMI was an independent factor influencing SVE prevalence. Naturally, the high prevalence of SVE can induce AF. Obesity is an independent risk factor for increasing the prevalence of AF (35). Although the pathophysiology of obesity implicating AF is not completely understood, the factors associated with it are as follows: genetic factors; clinical correlations such as hypertension, diabetes mellitus, and sleep apnea syndrome; coronary artery disease; ventricular adaptation; inflammation; oxidative stress; focal adrenergic pathways; and focal adiposity (36). Among these, epicardial focal adiposity has recently garnered much attention. Recent studies have reported that the increase in epicardial fat caused by obesity leads to the development of adipocyte infiltration into the myocardium, fibrosis, inflammation, oxidative stress, and impaired cardiac muscle activity in the myocardium (37). These factors can be triggers underlying the development of AF (37). Our findings show that a higher BMI contributes to the increased incidence of atrial premature complexes and may support recent findings on the role of obesity in AF.

### 4.6. Limitations

There are some limitations to this study. This study was performed for a brief duration of monitoring, i.e., a 24-hr period without any follow-up. We did not evaluate reproducibility between day-to-day values, which should be assessed using novel AECG devices, such as patch ECG, in the future (38). Moreover, the study population was restricted to individuals of Asian ethnicity; there is a possibility that the reference values of other ethnicities such as European, African, and Hispanic may be different. The minimum sample size required for the reference interval recommended by the Clinical and Laboratory Standards Institute guidelines (26) is met in this paper. However, a larger cohort and several follow-up recordings will be needed to investigate potential future directions of this work. In this study, the age range of 60–89 years was adopted as a single group. However, as shown in the meta-analysis by Williams et al. (27), the validity of the healthy value of 80 years of age and older is a controversial area and has not been clarified in previous reports. In order to verify the validity of using 60 years of age as a cutoff, we first compared the items listed as parameters in this study in the age group of 60–70 years and 70–89 years. There were no significant differences in all arrhythmia parameters (*P* = 0.204–0.916) except R-on-T, V3, and bradyarrhythmia. We then compared the parameters in the 60–75-year and 75–89-year age groups. There were no significant differences in any of the arrhythmia parameters except R-on-T, V3, and bradyarrhythmia (*P* = 0.349–0.972). These results support the fact that the age category of 60–89 years used in this study is valid. On the other hand, we could not validate R-on-T, V3, and bradyarrhythmia in the 60–89 years age group because the number of patients in all categories of R-on-T, V3, and bradyarrhythmia (Sinus pause and AV block) was less than 2, and statistics were difficult to obtain.

### 4.7. Conclusions

We presented age-specific reference values for AECG parameters derived from 24-h AECG in healthy individuals, over a wide age range (20–89 years). Notably, the reference values of VE and SVE were different in each generation. Moreover, we demonstrated that the incidence of VE was only related to the progression in age; hence, SVE was influenced by age and BMI increases, and RMSSD and HFnu decreases, which represent parasympathetic nervous system activity. This information will be useful for the diagnosis and prevention of diverse cardiac diseases in patients of various age groups in clinical settings. Future studies that account for the daily variance in healthy individuals are warranted to seek the reference interval of AECG.

## Data Availability

The raw data supporting the conclusions of this article will be made available by the authors, without undue reservation.
