# Supplementary material for: Age-related reference intervals for ambulatory electrocardiographic parameters in healthy individuals
Source: Front Cardiovasc Med. 2023 Mar 6;10:1099157. doi: 10.3389/fcvm.2023.1099157 (PMC10026132; doi:10.3389/fcvm.2023.1099157)
Supplement: Supplementary file 1 [file Datasheet1.docx]

Supplementary Material

Age-related Reference Intervals for Ambulatory Electrocardiographic Parameters in Healthy Individuals

Kenichi Hashimoto^*^, Naomi Harada, Motohiro Kimata, Yusuke Kawamura, Naoya Fujita, Akinori Sekizawa, Yosuke Ono, Yasuhiro Obuchi, Tadateru Takayama, Yuji Kasamaki, Yuji Tanaka

*** Correspondence:** Kenichi Hashimoto: hashimoto.kenich2@gmail.com

# Supplementary Tables

| Supplementary Table 1. Multiple regression analysis for log ventricular ectopy in individuals aged 20–39 years (n=120) | | | | | | | | |
| --- | --- | --- | --- | --- | --- | --- | --- | --- |
|  | R=0.417 | | |  | R=0.246* | | |  |
|  | β | p | VIF |  | β | p | VIF |  |
| Log VE | — | — | — |  | — | — | — |  |
| Age | 0.295 | 0.099 | 1.786 |  | 0.246 | 0.054 | 1.000 |  |
| Sex | 0.158 | 0.385 | 1.898 |  |  |  |  |  |
| SBP | 0.014 | 0.924 | 1.212 |  |  |  |  |  |
| BMI | 0.130 | 0.487 | 1.991 |  |  |  |  |  |
| E/e’ (septal) | -0.313 | 0.108 | 2.116 |  |  |  |  |  |
| E/e’ (lateral) | 0.264 | 0.188 | 2.273 |  |  |  |  |  |
| E/A | 0.179 | 0.229 | 1.261 |  |  |  |  |  |
| EF | 0.044 | 0.769 | 1.292 |  |  |  |  |  |
| Log SDNN | -0.176 | 0.425 | 2.787 |  |  |  |  |  |
| Log RMSSD | 0.036 | 0.931 | 9.843 |  |  |  |  |  |
| Log PNN50 | 0.145 | 0.694 | 7.772 |  |  |  |  |  |
| Log HFnu | -0.049 | 0.902 | 9.056 |  |  |  |  |  |
| Log LF/HF | -0.151 | 0.664 | 6.950 |  |  |  |  |  |
|  |  |  |  |  |  |  |  |  |
| EF=left ventricular ejection fraction, E/e’=early diastolic flow velocity/velocity of early diastolic mitral annular motion, HF=power in the high-frequency area, HFnu=HF normalized unit, LF/HF=power in the low-frequency/power in the high-frequency ratio, log VE=log ventricular ectopy, pNN50=percent of difference between adjacent normal RR intervals greater than 50 ms, RMSSD=root mean square successive difference, SBP=systolic blood pressure, SDNN=standard deviation of the mean normal RR intervals for 5-min segments (ms), VIF=variance inflation factor. *=Variables by multiple linear regression with back ward selection. (No significant factors influence to log VE by stepwise selection, then back war selection was performed) | | | | | | | |  |
|  |  |  |  |  |  |  |  |  |
|  |  |  |  |  |  |  |  |  |
|  |  |  |  |  |  |  |  |  |
|  |  |  |  |  |  |  |  |  |
|  |  |  |  |  |  |  |  |  |
|  |  |  |  |  |  |  |  |  |
|  |  |  |  |  |  |  |  |  |

| Supplementary Table 2. Multiple regression analysis for log ventricular ectopy in individuals aged 40–59 years (n=124) | | | | | | | |  |  |
| --- | --- | --- | --- | --- | --- | --- | --- | --- | --- |
|  | R=0.432 | | |  | R=0.189* | | | |  |
|  | β | p | VIF |  | β | p | VIF | |  |
| log VE | — | — | — |  | — | — | — | |  |
| Age | 0.134 | 0.290 | 1.335 |  | 0.189 | 0.079 | 1.000 | |  |
| Sex | 0.006 | 0.961 | 1.417 |  |  |  |  | |  |
| SBP | 0.126 | 0.357 | 1.559 |  |  |  |  | |  |
| BMI | -0.172 | 0.231 | 1.712 |  |  |  |  | |  |
| E/e’ (septal) | 0.343 | 0.056 | 2.642 |  |  |  |  | |  |
| E/e’ (lateral) | -0.206 | 0.232 | 2.478 |  |  |  |  | |  |
| E/A | 0.119 | 0.299 | 1.090 |  |  |  |  | |  |
| EF | 0.011 | 0.932 | 1.326 |  |  |  |  | |  |
| Log SDNN | -0.040 | 0.804 | 2.212 |  |  |  |  | |  |
| Log RMSSD | -0.009 | 0.979 | 9.829 |  |  |  |  | |  |
| Log PNN50 | 0.312 | 0.295 | 7.437 |  |  |  |  | |  |
| Log HFnu | -0.344 | 0.200 | 5.970 |  |  |  |  | |  |
| Log LF/HF | -0.105 | 0.625 | 3.849 |  |  |  |  | |  |
|  |  |  |  |  |  |  |  | |  |
| EF=left ventricular ejection fraction, E/e’=early diastolic flow velocity/velocity of early diastolic mitral annular motion, HF=power in the high-frequency area, HFnu=HF normalized unit, LF/HF=power in the low-frequency/power in the high-frequency ratio, log VE=log ventricular ectopy, pNN50=percent of difference between adjacent normal RR intervals greater than 50 ms, RMSSD=root mean square successive difference, SBP=systolic blood pressure, SDNN=standard deviation of the mean normal RR intervals for 5-min segments (ms), VIF=variance inflation factor. *=Variables by multiple linear regression with back ward selection. (No significant factors influence to log VE by stepwise selection, then back war selection was performed) | | | | | | | | |  |
|  |  |  |  |  |  |  |  |  |  |
|  |  |  |  |  |  |  |  |  |  |

| Supplementary Table 3. Multiple regression analysis for log ventricular ectopy in individuals aged 60–89 years (n=121) | | | | | | | | | |
| --- | --- | --- | --- | --- | --- | --- | --- | --- | --- |
|  | | R=0.359 | | |  | R=0.307* | | |  |
|  | β | | p | VIF |  | β | p | VIF |  |
| log VE | — | | — | — |  |  |  |  |  |
| Age | 0.178 | | 0.114 | 1.192 |  | 0.190 | 0.059 | 1.025 |  |
| Sex | -0.081 | | 0.516 | 1.469 |  |  |  |  |  |
| SBP | -0.007 | | 0.947 | 1.216 |  |  |  |  |  |
| BMI | 0.031 | | 0.783 | 1.247 |  |  |  |  |  |
| E/e’ (septal) | 0.219 | | 0.304 | 4.303 |  | 0.173 | 0.083 | 1.008 |  |
| E/e’ (lateral) | -0.037 | | 0.864 | 4.362 |  |  |  |  |  |
| E/A | 0.060 | | 0.574 | 1.078 |  |  |  |  |  |
| EF | -0.045 | | 0.680 | 1.152 |  |  |  |  |  |
| Log SDNN | -0.147 | | 0.266 | 1.651 |  |  |  |  |  |
| Log RMSSD | 0.175 | | 0.505 | 6.529 |  |  |  |  |  |
| Log PNN50 | -0.046 | | 0.840 | 5.043 |  |  |  |  |  |
| Log HFnu | — | | — | — |  | 0.304 | 0.085 | 3.164 |  |
| Log LF/HF | 0.111 | | 0.527 | 2.948 |  | 0.312 | 0.078 | 3.192 |  |
|  |  | |  |  |  |  |  |  |  |
| EF=left ventricular ejection fraction, E/e’=early diastolic flow velocity/velocity of early diastolic mitral annular motion, HF=power in the high-frequency area, HFnu=HF normalized unit, LF/HF=power in the low-frequency/power in the high-frequency ratio, log VE=log ventricular ectopy, pNN50=percent of difference between adjacent normal RR intervals greater than 50 ms, RMSSD=root mean square successive difference, SBP=systolic blood pressure, SDNN=standard deviation of the mean normal RR intervals for 5-min segments (ms), VIF=variance inflation factor, *=Variables by multiple linear regression with back ward selection. (No significant factors influence to log VE by stepwise selection, then back war selection was performed). Log HF nu was excluded from the Multiple regression analysis because of multicollinearity. | | | | | | | | |  |
|  |  |  |  |  |  |  |  |  |  |
|  |  |  |  |  |  |  |  |  |  |

| Supplementary Table 4. Multiple regression analysis for log supraventricular ectopy in individuals aged 20–39 years (n=120) | | | | | | | |  |  |
| --- | --- | --- | --- | --- | --- | --- | --- | --- | --- |
|  | R=0.499 | | |  | R=0.353^a^ | | | |  |
|  | β | p | VIF |  | β | p | VIF | |  |
| Log SVE | — | — | — |  | — | — | — | |  |
| Age | 0.219 | 0.040 | 1.492 |  | 0.294 | 0.001 | 1.015 | |  |
| Sex | -0.080 | 0.505 | 1.932 |  |  |  |  | |  |
| SBP | -0.164 | 0.169 | 1.885 |  |  |  |  | |  |
| BMI | -0.219 | 0.021 | 1.181 |  | -0.233 | 0.01 | 1.015 | |  |
| E/e’ (septal) | 0.060 | 0.646 | 2.306 |  |  |  |  | |  |
| E/e’ (lateral) | 0.170 | 0.199 | 2.321 |  |  |  |  | |  |
| E/A | -0.094 | 0.328 | 1.232 |  |  |  |  | |  |
| EF | 0.044 | 0.623 | 1.079 |  |  |  |  | |  |
| Log SDNN | -0.218 | 0.138 | 2.861 |  |  |  |  | |  |
| Log RMSSD | 0.668 | 0.013 | 9.402 |  |  |  |  | |  |
| Log PNN50 | -0.242 | 0.255 | 6.000 |  |  |  |  | |  |
| Log HFnu | -0.386 | 0.102 | 7.372 |  |  |  |  | |  |
| Log LF/HF | -0.088 | 0.673 | 5.852 |  |  |  |  | |  |
|  | | | |  |  |  |  | |  |
| EF=left ventricular ejection fraction, E/e’=early diastolic flow velocity/velocity of early diastolic mitral annular motion, HF=power in the high-frequency area, HFnu=HF normalized unit, LF/HF=power in the low-frequency/power in the high-frequency ratio, pNN50=percent of difference between adjacent normal RR intervals greater than 50 ms, RMSSD=root mean square successive difference, SBP=systolic blood pressure, SDNN=standard deviation of the mean normal RR intervals for 5-min segments (ms), VIF=variance inflation factor, log SVE=log supra ventricular ectopy. a. Variables by multiple linear regression with stepwise selection | | | | | | | | |  |
|  |  |  |  |  |  |  |  |  |  |

| Supplementary Table 5. Multiple regression analysis for log supraventricular ectopy in individuals aged 40–59 years (n=124) | | | | | | | | |
| --- | --- | --- | --- | --- | --- | --- | --- | --- |
|  | R=0.462 | | |  | R=0.271^a^ | | |  |
|  | β | p | VIF |  | β | p | VIF |  |
| log SVE | — | — | — |  | — | — | — |  |
| Age | 0.109 | 0.274 | 1.254 |  | 0.188 | 0.041 | 1.001 |  |
| Sex | -0.113 | 0.322 | 1.666 |  |  |  |  |  |
| SBP | -0.077 | 0.462 | 1.409 |  |  |  |  |  |
| BMI | -0.180 | 0.080 | 1.326 |  | -0.201 | 0.029 | 1.001 |  |
| E/e’ (septal) | 0.259 | 0.075 | 2.677 |  |  |  |  |  |
| E/e’ (lateral) | -0.134 | 0.355 | 2.675 |  |  |  |  |  |
| E/A | 0.225 | 0.152 | 1.067 |  |  |  |  |  |
| EF | 0.102 | 0.285 | 1.145 |  |  |  |  |  |
| Log SDNN | -0.041 | 0.740 | 1.910 |  |  |  |  |  |
| Log RMSSD | -0.267 | 0.273 | 7.524 |  |  |  |  |  |
| Log PNN50 | 0.387 | 0.089 | 6.508 |  |  |  |  |  |
| Log HFnu | -0.429 | 0.052 | 6.109 |  |  |  |  |  |
| Log LF/HF | -0.307 | 0.106 | 4.544 |  |  |  |  |  |
|  | | | |  |  |  |  |  |
| EF=left ventricular ejection fraction, E/e’=early diastolic flow velocity/velocity of early diastolic mitral annular motion, HF=power in the high-frequency area, HFnu=HF normalized unit, LF/HF=power in the low-frequency/power in the high-frequency ratio, pNN50=percent of difference between adjacent normal RR intervals greater than 50 ms, RMSSD=root mean square successive difference, SBP=systolic blood pressure, SDNN=standard deviation of the mean normal RR intervals for 5-min segments (ms), VIF=variance inflation factor, log SVE=log supra ventricular ectopy. a. Variables by multiple linear regression with stepwise selection | | | | | | | |  |
|  |  |  |  |  |  |  |  |  |

| Supplementary Table 6. Multiple regression analysis for log supraventricular ectopy in individuals aged 60–89 years (n=121) | | | | | | | | |
| --- | --- | --- | --- | --- | --- | --- | --- | --- |
|  | R=0.365 | | |  | R=0.271^a^ | | |  |
|  | β | p | VIF |  | β | p | VIF |  |
| log SVE | — | — | — |  | — | — | — |  |
| Age | 0.128 | 0.218 | 1.207 |  | 0.202 | 0.021 | 1.000 |  |
| Sex | 0.025 | 0.822 | 1.417 |  |  |  |  |  |
| SBP | -0.030 | 0.762 | 1.106 |  |  |  |  |  |
| BMI | 0.043 | 0.688 | 1.306 |  |  |  |  |  |
| E/e’ (septal) | -0.272 | 0.153 | 4.071 |  |  |  |  |  |
| E/e’ (lateral) | 0.211 | 0.265 | 4.053 |  |  |  |  |  |
| E/A | 0.036 | 0.715 | 1.114 |  |  |  |  |  |
| EF | 0.164 | 0.096 | 1.091 |  |  |  |  |  |
| Log SDNN | -0.101 | 0.396 | 1.601 |  |  |  |  |  |
| Log RMSSD | 0.293 | 0.240 | 7.010 |  | 0.325 | <0.001 | 1.000 |  |
| Log PNN50 | -0.034 | 0.880 | 5.608 |  |  |  |  |  |
| Log HFnu | — | — | — |  |  |  |  |  |
| Log LF/HF | -0.028 | 0.867 | 3.095 |  |  |  |  |  |
| EF=left ventricular ejection fraction, E/e’=early diastolic flow velocity/velocity of early diastolic mitral annular motion, HF=power in the high-frequency area, HFnu=HF normalized unit, LF/HF=power in the low-frequency/power in the high-frequency ratio, pNN50=percent of difference between adjacent normal RR intervals greater than 50 ms, RMSSD=root mean square successive difference, SBP=systolic blood pressure, SDNN=standard deviation of the mean normal RR intervals for 5-min segments (ms), VIF=variance inflation factor, log SVE=log supra ventricular ectopy. a. Variables by multiple linear regression with stepwise selection. Log HF nu was excluded from the Multiple regression analysis because of multicollinearity. | | | | | | | |  |
|  |  |  |  |  |  |  |  |  |
|  |  |  |  |  |  |  |  |  |
